# Health Tract, No. 104

**Published:** 1862-09

**Authors:** 


### SUNDAY DINNERS.

Many a man has the courage to march to the cannon's mouth, and yet fails to resist over-indulgence in eating. He who has an intellect peerless among the generation in which he lives, becomes an imbecile at the dinner-table. The great Jonathan Edwards endeavored for two years to eat only as much as would meet the wants of the system; but day after day he found himself conquered; day after day he made the same record of these attempts—"failure!" For two years he went to his meals each day, resolving he would not eat too much; for two years he came away from the table forced to confess his convictions that he had "exceeded." When he had eaten a decent dinner, his common sense told him to desist; but then his uncommon sense would step in and say: "I shall be somewhat faint if I leave off now." So he would not leave off, and "in three minutes afterward I am convinced of excess." If such great minds have so little control over their appetites, it can not be wondered at that the less gifted, that the masses should abandon themselves to over-indulgence in all their propensities. Excess in eating may be avoided by taking three regular meals a day (nothing between) in a private room—having such an amount sent as observation shows can be eaten, and still leave a desire for more. For fifteen years that was the practice of that beautiful character and eminent philanthropist, Amos Lawrence, of Boston. There is wisdom and health in the practice of some who habitually avoid eating meat of any kind every Friday. A better plan still, quite as sure of religious profit as of physical advantage, is to take nothing for breakfast or supper on the Sabbath-day but a piece of cold bread and butter, with a single cup of weak coffee or tea, or other hot drink, taking at one P.M. a single bowl of any kind of soup, with the crust of cold bread broken into it. This can be taken to the utmost amount desired, for the nutritious material in it is so small that the sense of oppression induced by an equal bulk of a promiscuous meal is not experienced, or if so, it is slight, momentary, and harmless. If such a system of eating were adopted in families on the Sabbath, taking not an atom of any thing between meals, an amount of human suffering and sickness would be prevented, which to the multitude would be absolutely incredible, could it be expressed in numbers. Let the reader try it for a single day on himself, and see if he will not have a feeling of wellness on that and the succeeding day, which is delicious in its physical results, and prevents the indecorous and overpowering sleepiness which is so antagonistic of the profitable and enjoyable service which is proper to the sanctuary. It was an expressive saying of that gifted and model minister and man, the Rev. Dr. James W. Alexander, that "too many of us, by reason of 'Sunday dinners,' were more like gorged anacondas than any thing else, and thus became totally unfit for the afternoon service." He who deprives the body of one day's rest in seven, a Divinity has ordered in wisdom and mercy, will always suffer for it sooner or later now and hereafter; so the man who gives the stomach no rest, never will live out his appointed time, and will be miserable while he does live.

